# An Stomatin, Prohibitin, Flotillin, and HflK/C-Domain Protein Required to Link the Phage-Shock Protein to the Membrane in *Bacillus subtilis*

**DOI:** 10.3389/fmicb.2021.754924

**Published:** 2021-10-28

**Authors:** Abigail Savietto Scholz, Sarah S. M. Baur, Diana Wolf, Marc Bramkamp

**Affiliations:** ^1^Institute for General Microbiology, Christian-Albrechts-Universität zu Kiel, Kiel, Germany; ^2^Faculty of Biology, Ludwig-Maximilians-Universität München, Munich, Germany; ^3^Institute of Microbiology, Technische Universität Dresden, Dresden, Germany

**Keywords:** YdjI, SPFH-domain proteins, phage-shock protein, cell envelope stress response, flotillins, *Bacillus subtilis*

## Abstract

Membrane surveillance and repair is of utmost importance to maintain cellular integrity and allow cellular life. Several systems detect cell envelope stress caused by antimicrobial compounds and abiotic stresses such as solvents, pH-changes and temperature in bacteria. Proteins containing an Stomatin, Prohibitin, Flotillin, and HflK/C (SPFH)-domain, including bacterial flotillins have been shown to be involved in membrane protection and membrane fluidity regulation. Here, we characterize a bacterial SPFH-domain protein, YdjI that is part of a stress induced complex in *Bacillus subtilis*. We show that YdjI is required to localize the ESCRT-III homolog PspA to the membrane with the help of two membrane integral proteins, YdjG/H. In contrast to classical flotillins, YdjI resides in fluid membrane regions and does not enrich in detergent resistant membrane fractions. However, similarly to FloA and FloT from *B. subtilis*, deletion of YdjI decreases membrane fluidity. Our data reveal a hardwired connection between phage shock response and SPFH proteins.

## Introduction

All living cells are surrounded by a membrane barrier that shields them from the outside environment. The cell membrane is a dynamic structure containing a lipid bilayer with membrane integral and associated proteins. An intact membrane is essential to adapt to a changing environment. Thus maintenance of the cell membrane functionality is critical for cell viability. In the model-organism *Bacillus subtilis*, phospholipids such as the neutral lipid phosphatidylethanolamine, the anionic phospholipids phosphatidylglycerol and cardiolipin are the major lipid species in the cell membrane (Nickels et al., 2017), while the membrane proteome comprises proteins with several different functions such as membrane synthesis, remodeling, energy metabolism, as well as transport and signaling.

In eukaryotes, membrane regions with altered lipid composition and ordering are associated with the generation of micro-heterogenous domains referred to as lipid rafts or functional membrane microdomains (FMMs) (Simons and Ikonen, 1997; Jacobson et al., 2007). Lipid rafts are considered to be enriched in certain lipids such as cholesterol and sphingolipids. This leads to a low membrane fluidity, and a liquid-ordered (Lo-phase) arrangement of phospholipids. Importantly, these domain structures are thought to be short lived and of small size around 10–200 nm (Yuan et al., 2002; Owen and Gaus, 2013; Nickels et al., 2017). Existence and formation of membrane rafts or FMMs are still controversially discussed. Proteins such as flotillins have been considered raft marker proteins (Salzer and Prohaska, 2001; Stuermer and Plattner, 2005), however their involvement in membrane organization remains largely elusive. Early models were based on a detergent extraction method that relied on cold solubilization using Triton X-100. Proteins and lipids were separated into soluble and insoluble fractions after this treatment and, hence, termed detergent resistant membranes (DRMs) and detergent soluble membranes (DSMs). After such a membrane fractionation, flotillins enrich in DRMs, but it is commonly accepted now that DRM extraction is an artificial procedure that does not reflect any native membrane organization (Brown, 2006). Therefore, the molecular role of flotillins in eukaryotic membrane organization remains unclear, despite growing evidence that they are involved in membrane organization and signaling processes.

As their eukaryotic counterparts, *B. subtilis* encodes two flotillin homologs termed FloA and FloT (Donovan and Bramkamp, 2009; Lopez and Kolter, 2010). Bacterial flotillins are thought to help in the spatial organization of the membrane. Deletion of flotillins in *B. subtilis* leads in to an increase in membrane rigidity and Lo regions coalesce into large areas (Bach and Bramkamp, 2013). However, the precise function of flotillins in the organization of FMMs in *B. subtilis* is still unsolved. *B. subtilis* FloA and FloT do not co-localize and form distinct clusters of approximately 100 nm spatially separated (Dempwolff et al., 2016). Recently, it was reported that the lack of flotillins and the subsequent decrease the membrane fluidity (Bach and Bramkamp, 2013) leads to a reduction of MreB-directed elongasome complex activity of the peptidoglycan synthesis machinery (Zielinska et al., 2020). Subcellular localization and molecular function of lateral membrane organization and membrane domains started to be unraveled and several phenotypes were linked to FMMs including biofilm formation, protein secretion, competence and cell morphology (Donovan and Bramkamp, 2009; Dempwolff et al., 2012a; Bach and Bramkamp, 2013; Mielich-Suss et al., 2013; Mielich-Suss and Lopez, 2015). Importantly, the organization, maintenance and function of FMMs in bacterial membranes is still elusive.

The two bacterial flotillins, FloA and FloT, are members of a widely conserved and ancient protein family called Stomatin, Prohibitin, Flotillin, and HflK/C (SPFH) domain proteins (Tavernarakis et al., 1999; Browman et al., 2007). SPFH proteins usually share a tripartite domain core structure with an N-terminal membrane anchor and variable heptad repeat-rich sequences that are predicted to form inter-and/or intramolecular coiled-coil structures, termed the flotillin domain (Langhorst et al., 2005; Rivera-Milla et al., 2006; Hinderhofer et al., 2009). The common SPFH-domain found in all flotillins is likely involved in oligomerization, but alone does not bind the membrane (Bach and Bramkamp, 2015).

**FIGURE 1:**
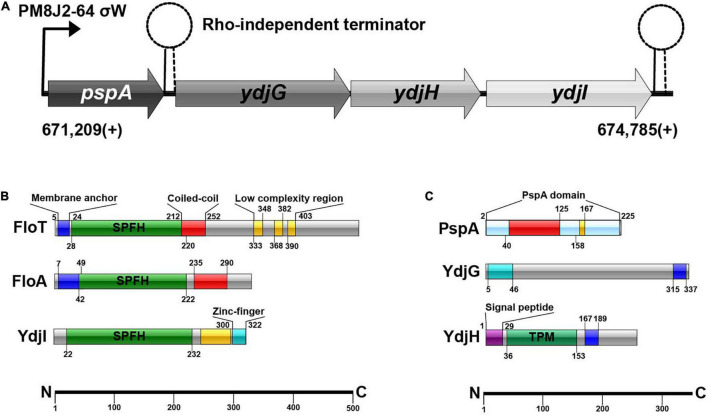
**(A)** Schematic overview of the *pspA*-*ydj*-operon. σ^W^-dependent promotor PM8J2-64 starts transcription at 671,209(+); transcription starts with *pspA*, continuing with *ydjG*, *ydjH* and subsequently *ydjI*. **(B)** SPFH-proteins in *B. subtilis* and their domains. **(C)** Proteins of the *ydj*-operon and their protein-domains.

Recently, a third protein containing the SPFH-domain, YdjI, was identified in *B. subtilis*. YdjI was considered to be a putative flotillin at first (Cozy et al., 2012; Popp et al., 2021; Ravi et al., 2021). Topological protein prediction, however, revealed that, unlike FloA and FloT, YdjI does not share the N-terminal transmembrane structure that anchors the protein to the membrane (Figure 1). Although, YdjI membrane association could be possible through positively charged residues in the N-terminus. When considering that the genetic organization and regulation of SPFH-domain proteins (including FloA and FloT) in *B. subtilis* is responsive to environmental stress, a plausible connection of SPFH-domain proteins in stress response pathways seems likely. Both FloA and FloT, are genetically regulated by an ECF sigma factor σ*^*w*^* (Huang et al., 1998; Wiegert et al., 2001), which is triggered by membrane stressors such as alkaline shock, high salt concentrations, and phage infection (Petersohn et al., 2001; Wiegert et al., 2001). Likewise, YdjI is part of the *pspA-ydjGHI* operon that is also regulated by σ*^*w*^* in *B. subtilis* (Wiegert et al., 2001; Cao et al., 2002). PspA and homologous proteins such as IM30/Vipp1 play an important role in cell envelope stress response, supporting membrane remodeling and stability (McDonald et al., 2015; Junglas et al., 2020a, b). They are originally defined as part of the phage shock protein family in *Escherichia coli* (Brissette et al., 1991; Kobayashi et al., 2007). PspA shares homology with conserved eukaryotic proteins including the mammalian ESCRT-III, functioning in stabilizing and remodeling membranes (McCullough et al., 2015), and VIPP1/IM30, essential for proper biogenesis of thylakoid membranes in chloroplasts and cyanobacteria (Manganelli and Gennaro, 2017). Just recently, the structures of several PspA/IM30/Vipp1 proteins have been resolved, confirming their structural similarity to the ESCRT-III proteins (Gupta et al., 2021; Junglas et al., 2021; Liu et al., 2021). In *B. subtilis*, PspA localizes to the membrane under stress conditions and protects the membrane against membrane-targeting antibiotics (Wolf et al., 2010; Kingston et al., 2013; Dominguez-Escobar et al., 2014; Popp et al., 2020). It has been speculated that the PspA/ESCRT-III system is an ancient, ubiquitous membrane repair system (Ravi et al., 2021).

Currently, our understanding of bacterial flotillins and their roles in bacterial physiology is rapidly evolving. The commonly accepted hypothesis was that bacterial SPFH-domain proteins reside in liquid-ordered membrane domains and act mainly as scaffold proteins to allow spatial and temporal membrane compartmentalization. However, further investigations from our group and others have pointed functions for the bacterial SPFH-domain proteins toward membrane modulation responsive to stress (Dempwolff et al., 2012b; Lee et al., 2012; Dempwolff and Graumann, 2014; Sawant et al., 2016; Zielinska et al., 2020).

Since the genetic regulation of FloT and YdjI is similar and stress related, we decided to further investigate the SPFH-domain protein YdjI in detail. Here, we describe the subcellular distribution of the *B. subtilis* YdjI protein and dissect the interaction with the other proteins encoded in the *pspA-ydjGHI* operon. We show that YdjI is required in the phage-shock protein response of *B. subtilis* and that the proteins encoded in the σ*^*w*^* controlled *pspA-ydjGHI* operon are highly interdependent for membrane localization. Contrary to the other SPFH-domain proteins in *B. subtilis* (FloA and FloT), YdjI does reside in fluid membrane region. Membrane complex formation of PspA depends on YdjI and the membrane integral proteins YdjG and YdjH. Unlike in *E. coli*, PspA focus formation and dynamics is independent of MreB in *B. subtilis*. Our data reveal a tight interaction network between YdjI, an SPFH-domain protein, and the phage-shock system in *B. subtilis*.

## Results

### YdjI Is an Stomatin, Prohibitin, Flotillin, and HflK/C-Domain Protein Localizing Into Liquid Disordered Membrane Regions

*Bacillus subtilis* 168 encodes a *pspA* (BSU_06180) homolog under control of a σ*^*w*^*-dependent promoter. Downstream of *pspA*, three structural genes, *ydjGHI* are positioned (Figure 1A). This operon is likely controlled via the promoter in front of *pspA*, however there is a Rho-independent terminator between *pspA* and *ydjI*. YdjI is a member of the SPFH-domain proteins. It shares the SPFH-domain with other bacterial flotillins, including FloA and FloT from *B. subtilis* (Figure 1B). Topology prediction analysis of YdjI suggests that the protein lacks a hydrophobic domain that could serve as a membrane anchor, consequently rendering YdjI soluble (Figure 1B). However, the two genes upstream of *ydjI*, *ydjG*, and *ydjH* encode for proteins that contain transmembrane helices and they are likely membrane integral proteins (Figure 1C). YdjI contains a low complexity region and a Zink-finger motif close to its C-terminus.

**FIGURE 2:**
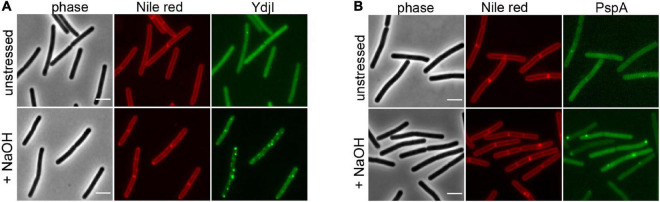
YdjI and PspA intracellular localization. Morphology of the exponentially growing strains labeled with Nile Red in unstressed (upper panel) and in alkali stress conditions (15 mM NaOH), lower panel. **(A)** YdjI-mNeonGreen and **(B)** PspA-GFP. Scale bars: 3 μm.

To study the subcellular localization of the *B. subtilis* YdjI protein, we constructed a translational YdjI-mNeonGreen fusion, replacing the native gene locus and, hence, expression of *ydjI-mNeonGreen* was still under native regulation. Furthermore, we used a PspA-GFP fusion to visualize PspA localization *in vivo*. PspA and YdjI were found to localize in discrete foci at the membrane of *B. subtilis* (Figures 2A,B). PspA expression was clearly enhanced under alkaline shock (Supplementary Figure 1), as expected since the promotor is known to be strongly induced by the ECF sigma factor σ^W^ (Huang et al., 1998; Wiegert et al., 2001). Western blotting or in-gel fluorescence revealed that PspA and YdjI fusion proteins were full length and only little degradation was observed (Supplementary Figure 1). While the cellular concentration of YdjI did not differ significantly under non-induced and stress induced conditions (stress was applied by addition of 15 mM NaOH), the concentration of PspA was greatly enhanced upon alkaline shock (Supplementary Figures 1A–C). These findings are in line with the presence of a Rho-independent terminator between *pspA* and *ydjG* (Figure 1A) leading to a substantial difference in the protein concentrations of PspA and YdjGHI after alkaline shock. The majority of YdjI was membrane associated, while the majority of PspA was found in the cytosol and not membrane associated (Supplementary Figures 1A–C). Although the PspA concentration was increased in the cell after alkaline shock, cell fractionation experiments show that the ratio of membrane associated and cytosolic PspA remained the same (Supplementary Figure 1C). Thus, PspA recruitment to the membrane was not increased after alkaline shock. The addition of 15 mM NaOH was sublethal for all individual deletion strains of *pspA* and the *ydj* genes tested here. In all cases addition of sodium hydroxide only marginally decreased growth rates compared to the unstressed control (Supplementary Figure 2). We conclude that alkaline shock with 15 mM NaOH does induce the *pspA-ydjFGHI* operon, but has no deleterious effect on the cells that might compromise localization studies.

**FIGURE 3:**
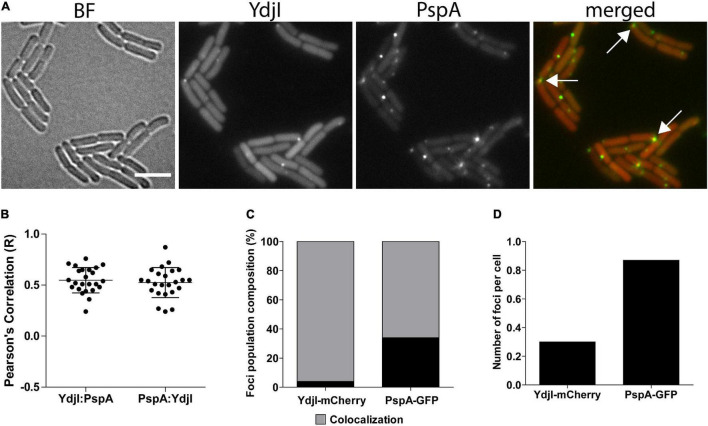
Co-localization analysis of PspA and YdjI proteins. **(A)** Micrographs showing exponentially growing cells of expressing YdjI-mCherry2 and PspA-GFP in alkali stress condition (15 mM NaOH). Arrows indicate co-localized foci. Scale bar: 4 μm. **(B)** Scattered dot plots of Pearson’s correlation (*R* values) assessed by Coloc-2 plugin from FIJI. Red to green (YdjI:PspA) and green to red (PspA:YdjI) co-localization analysis are shown. Error bars represent mean and standard deviation of values calculated for 24 cells. **(C)** Foci population composition in percentage of cells (*n* = 1650). **(D)** Ratio of total number of foci in each channel per total of cells (*n* = 1650).

In a next attempt we analyzed potential co-localization of PspA and YdjI. Both proteins formed membrane associated foci under stress conditions induced by 15 mM NaOH shock. We constructed a strain expressing PspA-GFP and YdjI-mCherry2 under control of their native promoters (strain ASB013). Microscopic examination of the cells revealed that PspA and YdjI readily form foci upon alkaline shock. The vast majority of YdjI foci co-localized with PspA foci (Figures 3A–C). Cells contained in average more PspA foci (Figure 3D and Table 1) and therefore not all PspA foci co-localized with a YdjI focus. However, a detailed analysis of co-localization using Pearson’s correlation revealed a high degree of co-localization for both proteins (Figure 3B).

**TABLE 1:** Number of PspA and YdjI foci per cell under alkaline shock and control conditions.

		**Average number of foci per cell (±SD)**	**No. of cells counted (*n*)**
PspA-GFP	NaOH	1.64 ± 0.18	497
	Control	0.49 ± 0.07	588
Δ*mreB;* PspA-GFP	NaOH	0.68 ± 0.07	539
	Control	0.21 ± 0.05	564
YdjI-mNG	NaOH	1.29 ± 0.05	629
	Control	0.71 ± 0.08	855
Δ*mreB;* YdjI-mNG	NaOH	0.81 ± 0.10	1015
	Control	0.49 ± 0.08	638

*Cells were grown in LB medium with supplements where necessary. Alkaline shock was induced by addition of 15 mM NaOH.*

**FIGURE 4:**
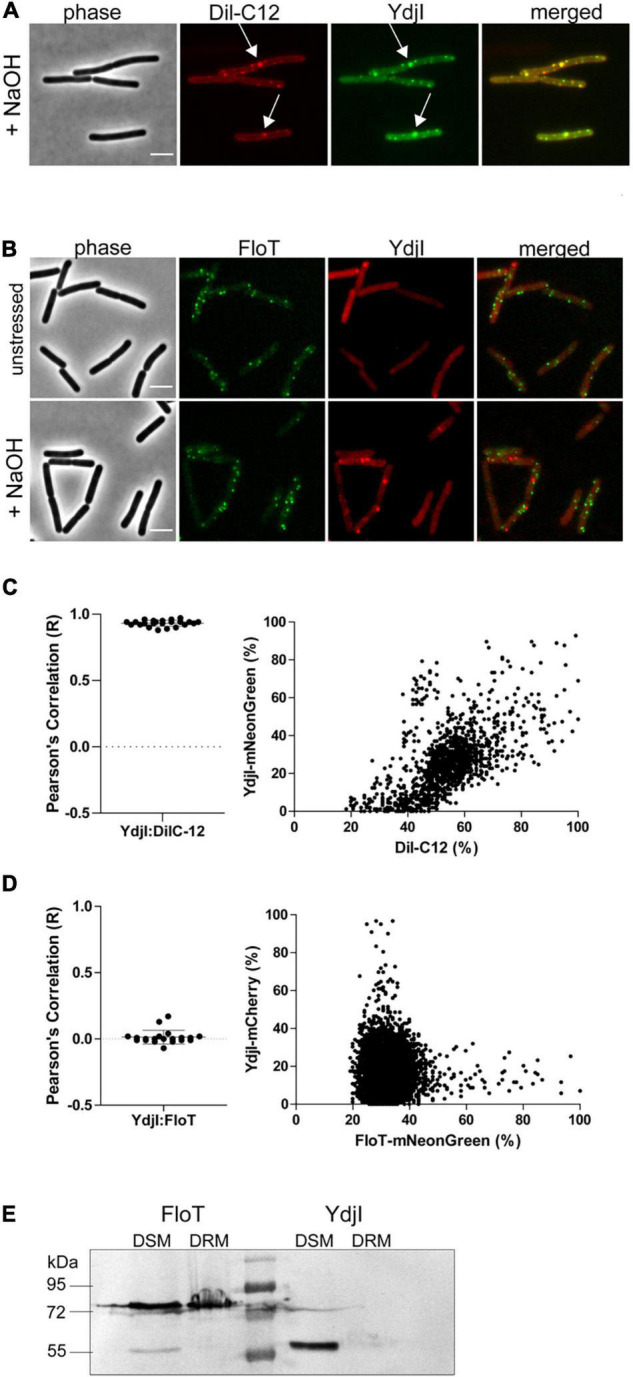
YdjI localizes into fluid membrane regions and does not co-localize with FloT. **(A)** YdjI co-localizes with fluid membrane regions as indicated by co-localization with Dil-C12 staining (arrows). **(B)** Localization of YdjI and FloT are independent. Micrograph show no localization of FloT and YdjI in a double tagged strain *floT-mNeonGreen; ydjI-mcherry*. Cells are in exponentially growing phase under unstressed or alkali stress conditions (15 mM NaOH). Scale bars: 3 μm. **(C)** Pearson’s co-localization analysis (*n* > 20) and fluorescence intensity correlation graphs (right panel) are shown for the YdjI and Dil-C12 localization. The graph displays a pixel by pixel intensity correlation between Dil-C12 and YdjI-mNeonGreen fluorescence. **(D)** Pearson’s co-localization analysis (*n* > 20) and fluorescence intensity correlation graphs are shown for the YdjI-mCherry and FloT-mNeonGreen localization. The graph on the right displays a pixel by pixel intensity correlation between FloT-mNeonGreen and YdjI-mCherry fluorescence. **(E)** Localization of YdjI in the detergent soluble membrane fraction (DSM). DSM/DRM extraction using strains expressing YdjI-mCherry (ASB049) and FloT-mCherry (ASB044) and subsequent immunoblotting using anti-mCherry antibodies is shown.

The subcellular localization pattern of YdjI and PspA was similar to the punctate membrane distribution of other bacterial SPFH-domain proteins, namely FloA and FloT (Donovan and Bramkamp, 2009; Lopez and Kolter, 2010). FloA and FloT were shown to localize into liquid ordered membrane regions (Bach and Bramkamp, 2013). We therefore wanted to address the preferred membrane region to which YdjI is recruited. To assess this, we performed *in vivo* co-localization studies of the YdjI-mNeonGreen fusion strain (strain ASB033) with Dil-C12, a well-known fluorescent probe for fluid membrane regions (Strahl et al., 2014). When *B. subtilis* cells were stained with Dil-C12, strictly punctuated fluorescent foci could be observed that overlapped significantly with the YdjI-mNeonGreen signal, with a Pearson’s correlation coefficient of 0.93 ± 0.022 (*n* = 23 cells) (Figures 4A,C). We also analyzed the co-localization pattern of FloT and YdjI by using a doubled tagged strain FloT-mNeonGreen; YdjI-mCherry2 (strain ASB154). In this strain both proteins were under control of their native promoter. YdjI did not co-localize with FloT in neither unstressed, or stressed conditions (Figures 4B,D). Fluorescence intensity correlation graphs for alkaline induced conditions in displaying a pixel by pixel correlation between Dil-C12 and YdjI-mNeonGreen (Figure 4C) or YdjI-mCherry2 and FloT-mNeonGreen (Figure 4D) revealed a clear anti-correlation between FloT and YdjI and a clear correlation between YdjI and Dil-C12.

Classical flotillins such as FloT can be enriched in so-called DRM extractions. We therefore aimed to compare the behavior of YdjI and FloT using DRM and DSM fractionation. Although, DRM/DSM fractionation is not suitable to make predictions about membrane domains in living cells, it allows the specific enrichment of proteins such as classical flotillins. Phase separation of DRM and DSM was achieved using a commercially available kit (see section “Materials and Methods”). Fractionation revealed that FloT is recovered in the DRM fraction as expected (Figure 4E). Although, part of the FloT protein was still found in the DSM fraction, a high proportion of the protein was recovered in the DRM fraction. On the contrary, YdjI was found exclusively in the DSM fraction (Figure 4E). This is an important difference, since currently, all SPFH-domain proteins have been associated with liquid ordered membrane regions and were thought to enrich in DRM fractions (Bramkamp and Lopez, 2015). In particular, the SPFH-domain itself was supposed to mediate contact and specificity to defined membrane regions. These data, together with the co-localization of YdjI with DilC-12 *in vivo*, confirm that YdjI behaves different compared to classical flotillins and likely resided in fluid membrane domains in the bacterial plasma membrane. Consequently, not all SPFH-domain proteins are associated with Lo domains.

### YdjI Is Required for PspA Focus Formation

**FIGURE 5:**
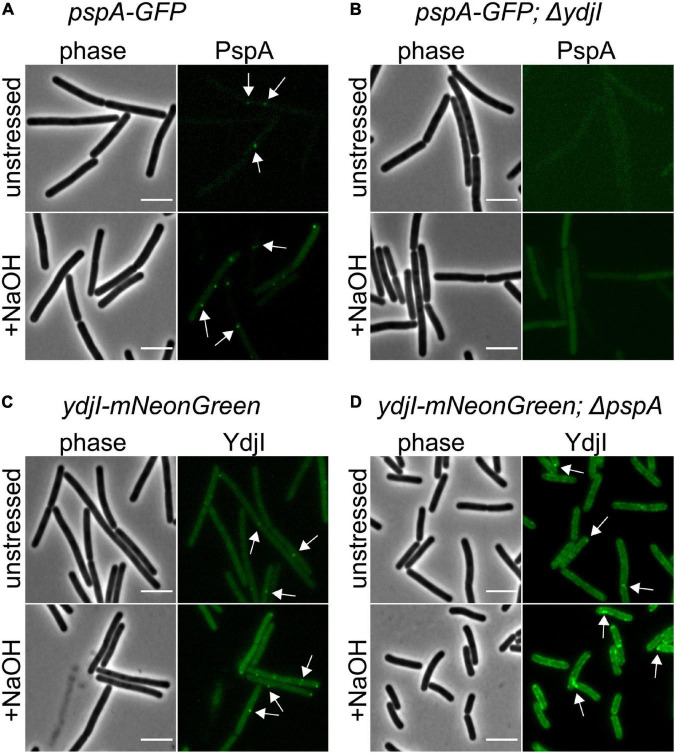
YdjI is required for PspA membrane anchoring and complex formation. **(A)** PspA-GFP foci are shown in unstressed and alkali stress conditions. **(B)** Deletion of *ydjI* delocalize PspA and the cells show no PspA foci in both conditions. Scale bars: 4 μm **(C)** *ydji-mNeonGreen* is shown under unstressed or stressed conditions (15 mM NaOH). **(D)** Deletion of *pspA* leads to YdjI overexpression. YdjI foci are still formed (arrows). Scale bars: 3 μm.

While FloA and FloT bind autonomously to the membrane, the subcellular localization of YdjI might require other proteins from the PspA-YdjGHI operon for membrane association, as hypothesized by the protein domain prediction analysis of YdjG and YdjH (Figure 1). To test this, we used YdjI-mNeonGreen and PspA-GFP fusion strains that lacked the *pspA* and *ydjI* gene, respectively, and analyzed the *in vivo* localization of the tagged proteins. The results revealed that PspA was not recruited to the membrane when YdjI was absent, regardless if the cells were induced or not with alkali-shock (Figures 5A–D), showing a strong dependency and interaction between PspA and YdjI for membrane complex formation. Importantly, ectopic expression of YdjI fully complemented this phenotype (Supplementary Figure 3). Deletion of *pspA* led to an increase of YdjI expression, due to the removal of the Rho-independent terminator in this strain. In this case we observed YdjI focus formation and conclude that YdjI complex formation does not require PspA (Figure 5D).

### YdjG and YdjH Are Part of the PspA-YdjI Interaction Network and Are Required for Membrane Localization of PspA and YdjI

**FIGURE 6:**
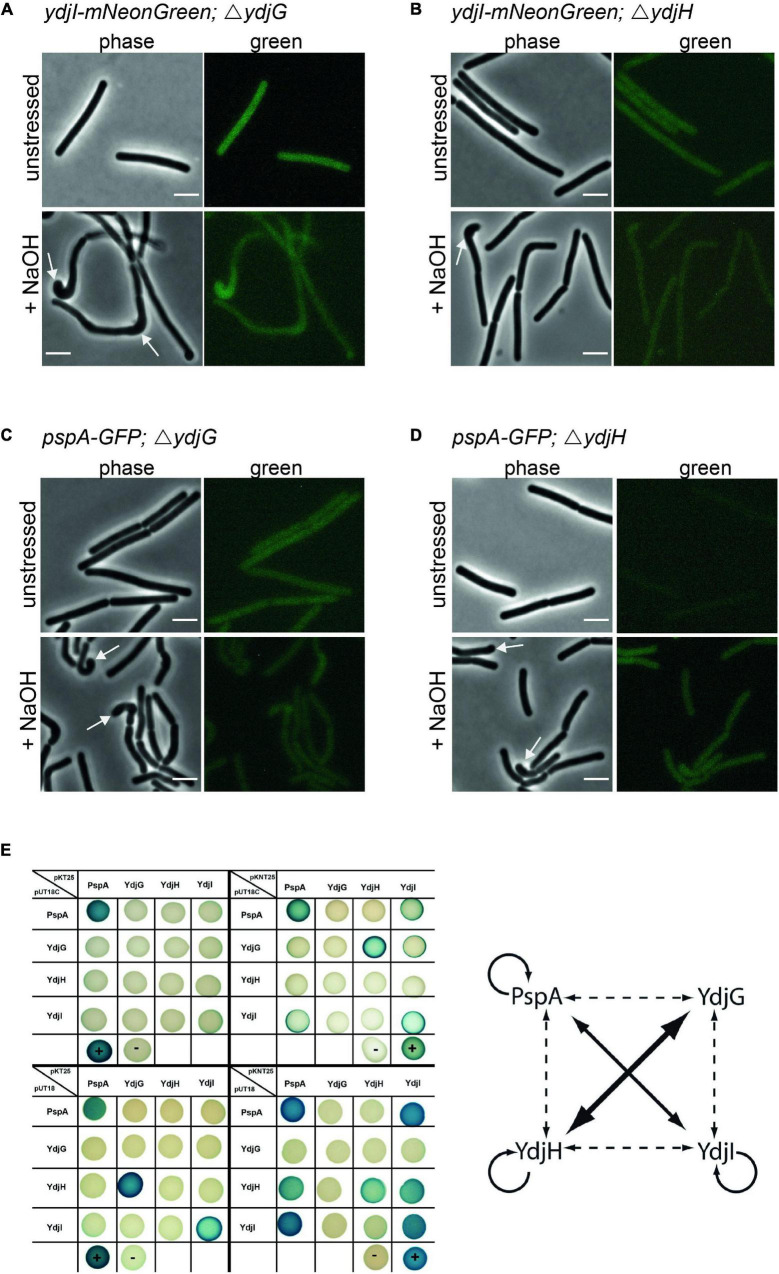
YdjG and YdjH serve as membrane anchor for PspA-YdjI protein complex and seem to have a role in cell-shape maintenance. Deletion of **(A)** *ydjG* and **(B)** *ydjH* delocalize YdjI foci formation under both unstressed and alkali stress conditions. The same is shown for PspA delocalization upon deletion of **(C)** *ydjG* and **(D)** *ydjH*. Arrows indicate a strong cell-shape defect upon deletion of either YdjG or YdjH, under stress conditions. Scale bars: 3 μm. **(E)** Interactions of proteins encoded in the *pspA-ydjGHI* operon via Bacterial-2-Hybrid. Blue colonies indicate protein–protein interaction, while in white colonies the target proteins do not interact. The positive and negative controls are marked by a plus minus, respectively. In pUT18C and pKT25 plasmids the T18 or T25 fragment of the adenylate cyclase is N-terminally fused to the protein of interest. pUT18 and pKNT25 encode for a C-terminal fusion to the protein of interest. The diagram represents a summary of the B2H analysis. The size of the arrow indicates the intensity of the blue color. Dashed lines indicate weaker protein–protein interactions.

Since YdjI is likely a soluble protein its membrane targeting should require an additional factor. We therefore reasoned that the two membrane proteins YdjG and YdjH that are encoded in the *pspA-ydj* operon are potential candidates for this membrane recruitment. Indeed, deletion of either *ydjG* or *ydjH* entirely abolished YdjI and PspA foci formation and membrane recruitment under normal growth and under alkali shock conditions (Figures 6A–D), hinting toward a direct interaction between the Ydj proteins. To test this hypothesis, we performed an extensive bacterial two hybrid interaction analysis. To this end, we fused the coding sequences of PspA, YdjI, YdjG, and YdjH to the split adenylate cyclase domains (Karimova et al., 1998). To minimize steric problems of the fusion proteins, we performed all possible combinations of N- and C-terminal fusions (Figure 6E). Using a blue-white screen on plate we observed a close interaction network between PspA and the Ydj proteins. PspA, YdjI and YdjH all showed self-interaction. We also detected a strong interaction between PspA and YdjI, corroborating the co-localization studies. Furthermore, we revealed an interaction of the two membrane components YdjG and YdjH with each other. Importantly, we also observed an interaction of YdjH with PspA and with YdjI, indicating that YdjH likely acts as the membrane anchor for the YdjI/PspA complex (Figure 6E).

### Absence of YdjG and YdjH Causes a Severe Phenotype Linked to Peptidoglycan Synthesis Machinery Delocalization

In strains deleted for either *ydjH* or *ydjG* we observed a severe cell morphological defect under alkali shock conditions. While unstressed cells lacking YdjG or YdjH reveal a normal morphology, these cells showed curved cells and swollen or bulged cell poles upon NaOH addition (Figures 6A–D). These aberrant morphologies are reminiscent of certain mutations known from components involved in cell wall synthesis such as a *gpsB* mutation (Claessen et al., 2008) or *mreBH* mutations (Carballido-Lopez et al., 2006). Therefore, we used fluorescent staining of nascent peptidoglycan synthesis, in order to visualize localization of the cell wall synthesis machinery. Strains with clean deletions of either *ydjG* and *ydjH* were stained with HADA (Kuru et al., 2015) and cells were stressed with NaOH (15 mM). HADA localizations were often found at the bulging cell poles and in areas were the cell body seems bent and misshaped (Supplementary Figures 4A,B). In wild type *B. subtilis* cells the cell poles are usually inert and do not localize active cell wall synthetic complexes. Cells lacking YdjG show a more pronounced phenotype including occasional branching (Supplementary Figure 4A). We conclude that YdjG might couple cell envelope stress signaling and cell wall synthesis. In absence of these proteins, cell wall synthesis becomes deregulated under stress conditions. Interestingly, deletion of YdjI or PspA did not exhibit this strong morphological phenotype and, hence mis-localization of the PspA/YdjI complex might not be the (sole) reason for this cell shape defect in an YdjG/H mutant.

### PspA and YdjI Foci Formation Does Not Depend on MreB

**FIGURE 7:**
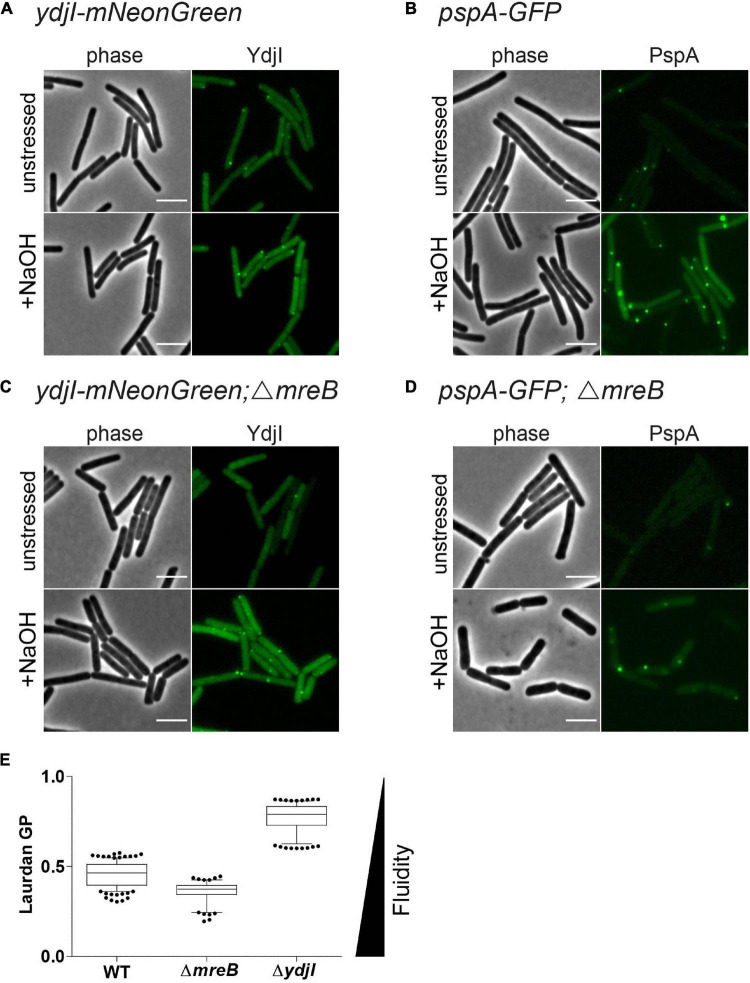
MreB is not required for YdjI and PspA foci formation. **(A)** YdjI localization in unstressed and alkaline stressed conditions. **(B)** PspA localization in unstressed and alkaline stressed conditions. **(C)** YdjI localization in a *mreB* mutant background. Note that in stressed and unstressed conditions YdjI foci are seen. **(D)** Upon deletion of the cytoskeletal protein *mreB*, PspA still localizes in membrane associated foci. Foci numbers in strains lacking *mreB* are reduced (see also Table 1). **(E)** Generalized polarization analysis after Laurdan staining of WT 168, MreB, and YdjI mutant strain are shown (*n* > 100); deletion of *mreB* causes increase in membrane fluidity, whereas YdjI deletion increases membrane rigidity. Scale bars 4 μm.

Anionic lipids and the cytoskeletal proteins MreB and RodZ define the spatio-temporal distribution and function of PspA in *E. coli* (Jovanovic et al., 2014). Based on these findings and the observed cell shape defect in *ydjG/H* mutants we wanted to analyze a potential involvement of the MreB cytoskeleton on PspA/YdjI complex formation. We constructed strains expressing YdjI-mNeonGreen and PspA-GFP in which we deleted the *mreB* gene (strains ASB155 and ASB123, respectively). In both strains we readily observed PspA and YdjI foci under alkali shock conditions, indicating that MreB is not essential for PspA or YdjI assembly (Figures 7A–D). However, a quantification of the PspA and YdjI foci per cell revealed that cells lacking MreB accumulate significant less foci compared to wild type cells (Table 1). While wild type cells had on average 1.6 PspA foci per cell, the *mreB* mutant strain had only 0.68 foci per cell. When cells were not stressed with sodium hydroxide we also observed a reduced number of PspA foci (0.21 foci in *mreB* mutant cells and 0.49 foci in wild type cells). Thus, absence of MreB leads to a 2.3-fold reduction of PspA foci formation under stressed and unstressed conditions. Similarly, the number of YdjI foci was reduced in absence of MreB in stressed and unstressed conditions (Table 1), lending support to the notion that PspA and YdjI foci formation is tightly connected. We next wanted to analyze whether PspA and YdjI foci are mobile in presence and absence of MreB. Time-lapse analysis revealed that in strains lacking *mreB* PspA and YdjI foci still retain their dynamic behavior (Supplementary Movies 1–8). Inspection of all movies revealed that many PspA and YdjI form foci that were mobile while others are rather static. A simple explanation might be that the static foci are acting on the membrane locally, maybe at damaged membrane areas. However, we did not observe a major difference in PspA or YdjI foci dynamics lacking MreB. We conclude that in *B. subtilis*, MreB is not essential for PspA and YdjI foci formation. However, deletion of MreB leads to altered foci numbers. We have shown that deletion of MreB led to a decrease in membrane fluidity (Figure 7E). A plausible explanation for the reduced foci number of PspA/YdjI might therefore a change in membrane fluidity.

### YdjI Fluidizes the Membrane, Similar to Flotillins

We have shown before that deletion of *mreB* or flotillins has an effect on membrane fluidity. Membrane fluidity can be experimentally addressed by using the generalized polarization (GP) values after a Laurdan staining. Laurdan intercalates into the membrane bilayer and its fluorescent spectra are sensitive to polarity and dipole dynamics. This allows a correlation of measured GP values and lipid packing in the membrane. Using Laurdan GP analysis we showed that deletion of *mreB* reduced the overall GP, rendering the membrane slightly more fluid. In contrast deletion of YdjI significantly increased the overall GP value. Thus, similar to the situation observed for the deletion of the flotillins FloA and FloT, deletion of the SPFH domain protein YdjI led to a rigidification of the bacterial membrane (Figure 7E).

## Discussion

Organization and surveillance of plasma membrane integrity is of vital importance for cellular life. Hence, several systems have been described that are part of membrane organization, stress perception and membrane repair (Willdigg and Helmann, 2021). Many of the involved proteins are ubiquitously distributed in all domains of life. One of the protein families that is widely distributed are the SPFH-domain proteins (Langhorst et al., 2005; Browman et al., 2007; Willdigg and Helmann, 2021). Among these proteins are flotillins, proteins that have been attributed with functional membrane domains in pro- and eukaryotes (Stuermer and Plattner, 2005; Bramkamp and Lopez, 2015). *B. subtilis* encodes two flotillin-like proteins, FloA and FloT (Bramkamp and Lopez, 2015). Both proteins are involved in membrane fluidity regulation (Bach and Bramkamp, 2013; Zielinska et al., 2020). *B. subtilis* encodes a third SPFH-domain protein, YdjI that is encoded in the *pspA-ydjGHI* operon (Popp et al., 2021; Ravi et al., 2021). We show here that YdjI is involved in membrane dynamics and PspA recruitment. The first protein encoded in the operon, PspA, is another ubiquitously found protein that is involved in cell envelope stress response. PspA forms large oligomeric complexes and shares a canonical ESCRT-III type fold (Gupta et al., 2021; Junglas et al., 2021; Liu et al., 2021). *In vitro* analysis shows that PspA proteins bind to membranes and can induce membrane deformation, fusion and fission events (Junglas et al., 2021). PspA homo-oligomeric complexes can be larger than 1 MDa and its donut-shaped arrangement imposes a positive curvature on the membrane (Junglas et al., 2021). Various PspA proteins, including IM30/Vipp1 from *Synechocystis* and PspA from *E. coli* have been shown to localize in foci along the membrane (Engl et al., 2009), similarly to the focus formation described here for the *B. subtilis* PspA and YdjI proteins. However, a main difference is that in *E. coli* the PspA foci are described to enrich at the cell poles and shuttle between poles (Engl et al., 2009), while for *B. subtilis*, we see less overall foci and not a strong bias for polar localization. Foci formation in diffraction limited fluorescence microscopy is in line with oligomerization, however the resolution is not high enough to judge about the molecular architecture. The precise nature of the YdjI or PspA foci remains to be elucidated, however, time-lapse analysis indicates that the complexes are dynamic. Occasionally, we observed static foci, potentially indicating action of YdjI/PspA foci at sites of membrane damage. So far, the best understood PspA system is that of *E. coli* in which PspA has been implicated with membrane repair and protection from proton leakage and subsequent dissipation of the proton motive force (Kleerebezem et al., 1996; Kobayashi et al., 2007). In *B. subtilis* two PspA homologs have been identified, LiaH and PspA. Both proteins are involved in response to cell membrane stress, for example induced by the pore forming antibiotic nisin (Wolf et al., 2010; Dominguez-Escobar et al., 2014). A common theme in PspA mediated stress response is the interaction of the PspA protein with a membrane receptor. In the *E. coli* system PspB and PspC recruit PspA to the membrane (Adams et al., 2003; Maxson and Darwin, 2006; Jovanovic et al., 2010). LiaH interacts with the membrane proteins LiaI and LiaG (Popp et al., 2021). Recent phylogenetic studies suggest a tight genomic connection of PspA with SPFH-domain (also termed Band-7) proteins (Popp et al., 2021; Ravi et al., 2021), however their functional interactions remained speculative. We show here, that the *B. subtilis* PspA requires the SPFH-domain protein YdjI for cluster formation and correct membrane recruitment. SPFH-domain proteins have been described as chaperones for complex formation, indicating a possible function in the oligomerization of PspA. This function is in line with our observation that stressed *B. subtilis* cells have in average more PspA cluster compared to YdjI and that almost all YdjI cluster co-localize with PspA. Thus, an initial interaction of these proteins for cluster formation and a subsequent release of PspA oligomers is plausible. Cluster formation also requires the two transmembrane proteins YdjG and YdjH. Absence of these proteins prevents PspA foci formation *in vivo* and an intricate interaction network has been revealed by bacterial two-hybrid analysis, extending recent studies (Popp et al., 2021; Ravi et al., 2021). Thus, all PspA systems studied so far require a membrane integral receptor complex. The interaction of PspA homologs and SPFH-domain proteins has also been described in other bacteria (Ravi et al., 2021). Although the precise function of SPFH-domain proteins in stress signal perception and membrane integrity is only partially understood, it emerges that membrane protection and surveillance systems are interlinked. Adding to this notion are also reports linking the functions of SPFH-domain proteins and bacterial dynamins (Dempwolff and Graumann, 2014). The latter are involved in membrane fusion and it was shown for the *B. subtilis* dynamin DynA that this protein catalyzes membrane fusion during nisin and phage stress (Burmann et al., 2011; Sawant et al., 2016), suggesting a complex membrane surveillance and repair network.

Stomatin, Prohibitin, Flotillin, and HflK/C-domain proteins that have been characterized so far localize to specific membrane regions (Bach and Bramkamp, 2013; Greenlee et al., 2021). Flotillins from eukaryotic cells have therefore been described as lipid raft marker (Dermine et al., 2001; Yokoyama and Matsui, 2020; Greenlee et al., 2021). Bacterial flotillins, such as FloA and FloT form specific clusters in the bacterial plasma membrane and both proteins are enriched in DRM fractions (Donovan and Bramkamp, 2009; Lopez and Kolter, 2010; Bach and Bramkamp, 2013). Surprisingly, we show here that YdjI localizes in focal assemblies in membrane regions of low fluidity. Consequently, we also recover YdjI only in DSM fractions. DRM fractionation is highly artificial and does not allow to draw conclusions on the native membrane organization. However, it is useful to enrich or separate certain membrane proteins based on the physical properties. Thus, the presence of the name giving SPFH-domain, does not predict *a priori* similar behavior of the proteins within the membrane. An obvious difference between FloA and FloT compared to YdjI is that the flotillins bind autonomously to the membrane by a transmembrane helix or hairpin-loop, respectively (Bach and Bramkamp, 2015). YdjI, in contrast, requires the membrane integral proteins YdjG and YdjH and transmembrane proteins might favor fluid membrane regions for insertion. So far it is unclear how the stimulus perception via YdjG/H is, but the observed knock-out phenotype of YdjG/H with a mis-localized cell wall synthesis, indicates that there is a tight connection of peptidoglycan synthesis and stress response. Despite the differences between FloA/T and YdjI, a remarkable similarity is the effect of *ydjI* deletion on membrane fluidity. Similar to a deletion of flotillins, loss of YdjI leads to an increase in the overall GP values, indicating that the protein is involved in membrane fluidization. It seems plausible that membrane fluidization might assist membrane repair because in fluid membranes fusion ad fission events occur with higher efficiency. Thus, bacterial cell membrane protection is using several combinations of PspA and SPFH-domain proteins that are hardwired in various combinations. Several of such systems can also act in one species, indicating that they either respond to specific signals or act as redundant systems. Given the importance of membrane integrity for cellular life, this is maybe not surprising.

## Materials and Methods

### *Bacillus subtilis* Strains and Growth Conditions

All *B. subtilis* strains used in this study are derived from strain 168 and are listed in Table 2. Construction of new strains was based on natural competence of *B. subtilis* (Harwood and Cutting, 1990). Gene integration or deletion was validated by colony PCR whereas the expression and localization of the fluorescent fusions were additionally validated by microscopy. Cells were grown in lysogeny broth (5 g/L yeast extract; 5 g/L NaCl; 10 g/L tryptone) at 37°C and 200 rpm. Induction of the *pspA* promoter was triggered by addition of 15 mM NaOH (Wiegert et al., 2001). Cell cultures were supplemented with spectinomycin (50 μg ml^–1^), chloramphenicol (5 μg ml^–1^), kanamycin (5 μg ml^–1^), erythromycin (1 μg ml^–1^), neomycin (10 μg ml^–1^), magnesium chloride (MgCl_2_, 25 mM), and/or sucrose (0.3 M) when necessary.

**TABLE 2:** Strains used in this study.

**Strain**	**Genotype**	**Reference/Source**
***Escherichia coli***		
BTH101	*E. coli F-, cya-99, araD139, galE15, galK16, rpsL1 (str^R^), hsdR2, mcrA1, mcrB1*	BACTH System Kit (Euromedex)
DH5α	*E. coli F–*Φ80lacZM15 *(lacZYAargF) U169 recA1 endA1 hsdR17(rk−, mk*+) *phoA supE44 thi-1 gyrA96 relA1*λ	Invitrogen/Thermo Fisher
NEB Turbo	*F*′ *proA*+ *B*+ *lacIq*Δ *lacZ M15/fhuA2*Δ*(lac-proAB) glnV gal R(zgb210::Tn10)TetS endA1 thi-1*Δ*(hsdS-mcrB)5*	New England Biolabs
25 N F	NEB5α pKNT25-*pspA*	This work
25 N G	NEB5α pKNT25-*ydjG*	This work
25 N H	NEB5α pKNT25-*ydjH*	This work
25 N I	NEB5α pKNT25-*ydjI*	This work
25 C F	NEB5α pKT25-*pspA*	This work
25 C G	NEB5α pKT25-*ydjG*	This work
25 C H	NEB5α pKT25-*ydjH*	This work
25 C I	NEB5α pKT25-*ydjI*	This work
18 N F	NEB5α pUT18-*pspA*	This work
18 N G	NEB5α pUT18-*ydjG*	This work
18 N H	NEB5α pUT18-*ydjH*	This work
18 N I	NEB5α pUT18-*ydjI*	This work
18 C F	NEB5α pUT18C-*pspA*	This work
18 C G	NEB5α pUT18C-ydjG	This work
**Bacillus subtilis**		
ASB001	trpC2 *Bacillus subtilis*, wild type 168	Laboratory collection
ASB013	trpC2 pspA:pspA-GFP; ydjI:ydjI-mCherry (cat)	This work
ASB023	trpC2 floT::floT-mNeonGreen (spc)	This work
ASB033	trpC2 ydji::ydjI-mNeonGreen (cat)	This work
ASB044	trpC2 floT:: floT-PAmCherry (cat)	This work
ASB049	trpC2 ydjI::ydjI-mCherry2 (cat)	Lab collection
ASB053	trpC2 ydjI::kan	BGSC Knockout collection
ASB054	trpC2 ydjG::kan	BGSC Knockout collection
ASB055	trpC2 ydjH::kan	BGSC Knockout collection
ASB083	trpC2 pspA::kan	BGSC Knockout collection
ASB094	trpC2 mreB::kan amyE::spc Pxyl mrfpRuby-mreB	Zielinska et al., 2020
ASB122	trpC2 mreB::neo	Formstone and Errington, 2005
TSB2351	trpC2 pspA::pspA-GFP (C-term fusion) markerless	Thorsten Mascher/Annika Sprenger (Dresden)
ASB153	trpC2 ydji::ydjI-mNeonGreen cat; pspA::kan	This work (ASB083→ASB033)
ASB154	trpC2 ydjI:ydjI-mCherry2 (cat); floT::floT-mNeonGreen (spc)	This work (ASB023→ASB049)
ASB155	trpC2 ydji::ydjI-mNeonGreen cat; mreB::neo	This work (ASB033→ASB122)
ASB156	trpC2 pspA::pspA-GFP; ydjG::kan	This work (TSB2351→ASB054)
ASB157	trpC2 pspA::pspA-GFP; ydjH::kan	This work (TSB2351→ASB055)
ASB159	trpC2 ydji::ydjI-mNeonGreen cat; ydjG::kan	This work (ASB033→ASB054)
ASB160	trpC2 ydji::ydjI-mNeonGreen cat; ydjH::kan	This work (ASB033→ASB055)
ASB162	trpC2 pspA::pspA-GFP; ydjI::kan	This work (TSB2351→ASB053)
ASB166	trpC2 mreB::neo pspA::pspA-GFP	This work (ASB122→TSB2351)
ASB170	trpC2 pspA::pspA-GFP ydjI::kan amyE::ydjI	This work

*Kan = kanamycin; Cat = chloramphenicol; Neo = neomycin; Spc = spectinomycin; Tet = tetracycline.*

### Strain Constructions

Plasmids were constructed using standard cloning techniques and Golden Gate Cloning Assembly (Engler et al., 2009). For DNA amplification via PCR, Phusion DNA polymerase was used. Restriction enzymes were purchased from New England Biolabs (NEB, Ipswich, MA, United States) and used accordingly with their respective protocols. *E. coli* colonies were checked by colony PCR, using EconoTaq polymerase (Lucigen) and all plasmids were verified by sequencing. All strains, primers and plasmids are listed in Tables 2–4, respectively. Plasmid cloning was carried out in *E. coli* DH5α or in DH5α Turbo (New England Biolabs). The strains FloT-PAmCherry and YdjI-mNeonGreen were constructed as follows: PAmCherry fragment was amplified from the plasmid pPAmCherry1-N1 using the primers pamCherry-F and pamCherry-R. The fragment of mNeonGreen was amplified from pNCS-mNeonGreen plasmid using the primers mNeonGreen-F and mNeonGreen-R. The *Bsa*I site in pUC18 plasmid was mutated using the primers pUC18mut-F and pUC18mut-R resulting in the plasmid pUC18mut. YdjI and FloT including their endogenous promoter and ribosomal binding site were amplified from genomic *B. subtilis* DNA using the primers pairs YdjI-GG-F/YdjI-GG-R and FloT-GG-F/FloT-GG-R and cloned into pUC18mut. FloT-PAmCherry construct was done by Golden Gate assembly of the fragments yuaGIN, PAmCherry, cat, yuaGDown and pUC18BsaI, respectively resulting in the plasmid pUC18mut-yuaGIN-PAmCherry-cat-yuaGDown. For the ectopic expression of *ydjI*, we constructed plasmid pJPR1-ydjI. The coding sequence of *ydjI* including its own ribosomal binding site was amplified from genomic *B. subtilis* DNA using the primer pair *Xba*I_RSB_ydjI_UP and *Eag*I_Stop_ydjI_DOWN and subsequently cloned into pJPR1. The final plasmid was transformed into *B. subtilis* and integration into the *amyE* locus was confirmed.

All oligonucleotides resulting in the respective fragments can be found in Table 4. All plasmids were transformed into *B. subtilis* was based on natural competence of *B. subtilis* (Harwood and Cutting, 1990) and screened for double cross over. Gene integration or deletion was validated by colony PCR whereas the expression and localization of the fluorescent fusions was additionally validated by microscopy.

**TABLE 3:** Plasmids used in this study.

**Name/Number**	**Genotype or Description**	**Reference/Source**
pUT18C	P*lac*, T18 (AA 225 to 399 of ccaA), MCS, *bla*, Ori ColE1	Karimova et al., 1998
pUT18	P*lac*, MCS, T18 (AA 225 to 399 of ccaA), *bla*, Ori ColE1	Karimova et al., 1998
pUT18C-zip	P*lac*, T18 (AA 225 to 399 of ccaA)-leucine zipper of GCN4, MCS, *bla*, Ori ColE1	Karimova et al., 1998
pKNT25-zip	P*lac*, T25 (first 224 AA of ccaA)-leucine zipper of GCN4, MCS, *kan*, Ori p15A	Karimova et al., 1998
pKT25	P*lac*, T25 (first 224 AA of ccaA), MCS, *kan*, Ori p15A	Karimova et al., 1998
pKNT25	P*lac*, MCS T25 (first 224 AA of ccaA) *kan*, Ori p15A	Karimova et al., 1998
pUC18	*OriColE1* MCS CbR; *bla lacZ*	(Yanisch-Perron et al., 1985) Thermo Fisher Scientific
pUC18mut	pUC18 mutated MCS, *bla lacZ*	This work
pPAmCherry1-N1	Amplification of PAmCherry fragment	Subach et al., 2009
pNCS-mNeonGreen	Amplification of mNeonGreen fragment	Shaner et al., 2013
pJPR1-ydjI	*bla amyE3*′ *cat Pxyl ydjI amyE5*′	This work
pUC18mut-yuaGIN-PAmCherry-cat-yuaGDown	*bla floTIn-PAmCherry-cat-floTDown*	This work
25 N F	pKNT25-*pspA*	This work
25 N G	pKNT25-*ydjG*	This work
25 N H	pKNT25-*ydjH*	This work
25 N I	pKNT25-*ydjI*	This work
25 N F	pKNT25-*pspA*	This work
25 N G	pKNT25-*ydjG*	This work
25 N H	pKNT25-*ydjH*	This work
25 N I	pKNT25-*ydjI*	This work
18 N F	pUT18-*pspA*	This work
18 N G	pUT18-*ydjG*	This work
18 N H	pUT18-*ydjH*	This work
18 N I	pUT18-*ydjI*	This work
18 C F	pUT18C-*pspA*	This work
18 C G	pUT18C-*ydjG*	This work
18 C H	pUT18C-*ydjH*	This work
18 C I	pUT18C-*ydjI*	This work

**TABLE 4:** Oligonucleotides used in this study.

**Name**	**Sequence**	**Restriction site**
pUC18mut-F	TTTGGTCTCAGGTTCTCGCGGTATCATTGCAGC	*Bsa*I
pUC18mut-R	TTTGGTCTCAAACCACGCTCACCGGCTCCAG	*Bsa*I
pUC18BsaI-F	GTCGGTCTCAACTAGAATTCGTAATCATG	*Bsa*I
pUC18BsaI-R	CTCGGTCTCATCGGAAGCTTGGCACTGGC	*Bsa*I
pamCherry-F	AATGGTCTCTGGAGGGATGGTGAGCAAGGGCGAGGA	*Bsa*I
pamCherry-R	TTTGGTCTCGCGAATTACTTGTACAGCTC	*Bsa*I
mNeonGreen-F	AATGGTCTCTGGAGGGATGGTGAGCAAGGGCGAGGA	*Bsa*I
mNeonGreen-R	TTTGGTCTCGCGAATTACTTGTACAG CTC	*Bsa*I
mCherry2-F	AACCTCCGGTCTCCAATGGTCAGCAAGGGAGAG	*Bsa*I
mCherry2-R	AACCTCCGGTCTCCAGGATCCTGAGCCGCTTC	*Bsa*I
Cat-GG-F	GGAGGTCTCTTTCGGGCTTTAGATAAAAATTTAGGAGGC	*Bsa*I
Cat-GG-R	CACGGTCTCCCATTTTATAAAAGCCAGTC	*Bsa*I
Spc-GG-F	GGAGGTCTCTTTCGGGGTGAAAGGATGTACTTA	*Bsa*I
Spc-GG-R	CACGGTCTCCCATTTAATTGAGAGAAGTT	*Bsa*I
*ydjI*-GG-F	CCCTTTAATTATCCTCAAGAGG	*Bsa*I
*ydjI*-GG-R	TATGGTCTCCCTCCTACAAGCTTCTGGCC	*Bsa*I
*yuaG*IN-GG-F	CTAGGTCTCTCCGAGCCGATGCCAAGAAG	*Bsa*I
*yuaG*INGG-R	TATGGTCTCCCTCCCTCTGATTTTTGGAT	*Bsa*I
*yuaG*DownGG-F	ACGGGTCTCAAATGGGAAAGGGCAGAACCGTATGGT	
*yuaG*DownGG-R	CGGGGTCTCTTAGTTTTCAGGTGAATAGG	
*pspA*-F-*Bam*HI	AAAGGATCCAATGATGATAATTGGAAGATTTAAAGATATTATG	*Bam*HI
*pspA*-R-*Kpn*I-stop	AAAGGTACCTTACTTATCGAGCATCATTTTCG	*Kpn*I
*pspA*-R-*Kpn*I	AAAGGTACCTTATCGAGCATCATTTTCG	*Kpn*I
*ydjG*-F-*Bam*HI	AAAGGATCCAATGATAATATCTTATAAGTGTCCGAACTGC	*Bam*HI
*ydjG*-R-*Eco*RI-stop	AAAGAATTCTCAAAATCCGCCTCCCATC	*Eco*RI
*ydjG*-R-*Eco*RI	AAAGAATTCACAAATCCGCCTCCCATC	*Eco*RI
*ydjH*-F-*Bam*HI	AAAGGATCCAATGCGTGGATTTTTTGGG	Bam
*ydjH*-R-*Eco*RI-stop	AAAGAATTCTTAAAAACTGCCCCGGCTCC	*Eco*RI
*ydjH*-F-*Eco*RI	AAAGAATTCCCAAAACTGCCCCGGCTCC	*Eco*RI
*ydjI*-F-*Bam*HI	AAAGGATCCAATGTCGTTTTTCAGAAATCAATTAG	*Bam*HI
*ydjI*-R-*Kpn*I-stop	AAAGGTACCTTATACAAGCTTCTGGCCG	*Kpn*I
*ydjI*-R-*Kpn*I	AAAGGTACCGCTACAAGCTTCTGGCCGC	*Kpn*I
*Xba*I_RSB_ydjI_UP	GATTCTAGAATAGAGAAAGGGAGAG	*Xba*I
*Eag*I_Stop_ydjI_DOWN	ATCCGGCCGGTATGATTCTTATACAAGC	*Eag*I

### Bacterial 2-Hybrid Analysis

Bacterial adenylate cyclase-based two-hybrid (BACTH) assay was performed according the protocol described by Karimova et al. (1998). Strain *E. coli* BTH101 (Euromedex) was co-transformed with plasmids containing genes of interest fused either to T18 or T25 fragments of adenylate cyclase. The adenylate cyclase is divided into two parts, each of them either N- or C-terminal present on the vectors pUT18/pKT25N or pKT25/pUT18C, respectively. Since the interaction of the proteins can be influenced by the position of the adenylate cyclase domains, all plasmid combinations were used (Table 3). After transformation, 10 μl of the cell suspensions were spotted on agar plates containing ampicillin (100 μg ml^–1^), kanamycin (50 μg ml^–1^), IPTG (0.5 mM), and X-Gal (40 μg ml^–1^) and incubated at 37°C for approximately 40 h. Blue colonies indicate potential protein-protein interactions. Empty vectors or vectors containing in frame fusions of the leucine zipper of GCN4 to the T18/T25 fragments were used as negative and positive controls, respectively. The plates were stored at 4°C protected from light for at least 4 days to increase contrast and intensity of the colony color.

### Detergent Soluble Membrane and Detergent Resistant Membrane Isolation

Detergent resistant membrane and detergent soluble membrane fractions were separated as described previously by Salzer and Prohaska (2001) with modifications. To this end, the kit CelLytic^*TM*^ MEM Protein Extraction Kit from Sigma-Aldrich was used. Overnight cultures of strains YdjI-mCherry2 and FloT-PAmCherry were diluted (1:100) into 50 ml of LB medium and incubated at 37°C (200 rpm) until an OD_600__nm_ between 0.5 and 0.8 was reached. The cells were induced with 15 mM NaOH (final concentration) and incubated for an additional hour under the same conditions. The cells were washed in cold saline phosphate buffer containing 1 mM EDTA and concentrated to a final volume of 1.75 ml. 10 μl of the provided protease inhibitor cocktail was added and the cells were cracked with 7 cycles of sonication (30% amplitude, 0.5 s/pulse ON, 0.5 s/pulse OFF, 30 s total), waiting on ice between the runs. The cell debris was pelleted with a bench top centrifuge (18,000 × *g*, 10 min, 4°C) and 1 ml of the supernatant was transferred into ultracentrifugation tubes. Membranes were pelleted at 235,000 × *g* for 40 min at 4°C. The membrane pellet was resuspended in 600 μl lysis and separation buffer supplemented with protease inhibitor cocktail, and incubated for 10 min on ice. The suspension was centrifuged for an additional 5 min (4°C, 18,000 × *g*). For the separation of the DSM and the DRM fraction the supernatant was heated at 30°C for 3–5 min. Both layers were separated at 3000 × g for 3 min (>20°C). Samples were taken from the upper hydrophilic phase (DSM) and from the lower hydrophilic phase (DRM) and used in Western-blot downstream analysis.

### Sample Preparation and Western-Blot Analysis

Overnight cultures containing the appropriate antibiotic and/or supplements were diluted 1:100, incubated at 37°C and induced with 15 mM NaoH when an OD_600__nm_ between 0.5 and 0.8 was reached. Controls were monitored so that they did not exceed mid-log phase. After 1 h induction, cell densities were normalized, pelleted in a bench centrifuge (max. speed for 10 min, 4°C), and washed with PBS supplemented with 1 mM EDTA. Cell pellets were stored at −80°C for further use. For separation into cytosol and membrane cell pellets, the samples were resuspended in 500 μl of the same buffer (PBS + 1 mM EDTA), supplemented with protease inhibitor cocktail (Sigma Aldrich). The suspensions were homogenized (7 cycles of sonication at 30% amplitude, 0.5 s/pulse ON, 0.5 s/pulse OFF, 30 s total). The cell debris were pelleted in a bench centrifuge (18,000 × *g*, 10 min, 4°C) and 500 μl of the supernatant was transferred into ultracentrifuge tubes. Pellets were washed once and resuspended in same volume as before. Samples of cytosol and membrane were incubated for 20 min at room temperature with 4× SDS loading dye. Samples were subjected to SDS-PAGE through a 10 or 12 % gel and blotted onto a PVDF membrane (Bio-Rad) at 200 mA for 2 h and blocked overnight with 5% milk solution in buffer (50 mM Tris–HCl pH 7.5/150 mM NaCl) supplemented with or without Triton X 100 under slight agitation at 4°C. The blots were incubated with anti-α-mCherry (1:1000) or, anti-mNeonGreen (1:1000) antibodies and incubated at room temperature for at least 1 h with mild shaking. The blots were then washed five times with buffer and incubated with the corresponding secondary antibody (anti-rabbit IgG for α-mCherry and anti-α-GFP; anti-mouse IgG for anti-mNeonGreen) conjugated with the corresponding alkaline phosphatase (1:2000) or horse radish peroxidase conjugate (1:5000, Invitrogen). Immunoblots were again washed with buffer and developed with NBT/BCIP or Pierce^*TM*^ ECL Western Blotting Substrate (Thermo Scientific) using the Chemi Hi Sensitivity program with signal accumulation of the ChemiDoc^*TM*^ MP. For in-gel fluorescence of GFP fusion proteins, SDS-PAGE gels were rinsed in distilled water and subsequently subjected to Blue Epi illumination in the ChemiDoc^*TM*^ MP. Emission was detected by a 530/28 filter. For in-gel fluorescence of Δ*mreB pspA*-*GFP* cultures were sonicated and directly applied to acrylamide gel-electrophoresis. Fluorescence of GFP was visualized in gels using a Bio-Rad ChemiDoc imager.

### Sample Preparation for Microscopy

Overnight cultures of the strains were inoculated 1:100 into LB medium with supplements if necessary and grown to OD_600__nm_ 0.5–0.8. To induce PspA promoter, alkaline shock condition (15 mM NaOH, final concentration) was added. Cells were washed and resuspended in medium supplemented, when appropriate, with Dil-C12 solution (2.5 μg ml^–1^, final concentration) for staining of fluid membrane regions as described before (Strahl et al., 2014), or with HADA (Kuru et al., 2015), as a peptidoglycan synthesis machinery probe. Exponentially growing cells were immobilized on 1% agarose-pads (w/v) in the appropriate medium mounted onto microscope slides using Gene Frames (1.0 × 1.0 cm chamber, 0.25 mm thickness, from Thermo Scientific) as described before (de Jong et al., 2011). Samples were covered with high precision microscopy cover glasses (170 ± 5 μm, Paul Marienfeld GmbH & Co. KG, Germany) and directly taken for microscopic observations.

### Fluorescence Microscopy

Standard fluorescence microscopy was carried out using an Axio Zeiss Imager M1 fluorescence microscope (EC Plan-Neofluar 100×/1.30 Oil Ph3 objective) equipped with an AxioCam HRm camera. For membrane fluidity experiments, a Delta Vision Elite microscope (Applied Precision, GE Healthcare) equipped with an Insight SSI Illumination, an X4 Laser module, a CoolSnap HQ CCD camera and a temperature-controlled chamber set up at 37°C was used. Laurdan images were taken with an Olympus UplanSApo 100×/1.4 oil objective. Data processing was performed with SoftWoRx Suite 2.0 and FIJI Software’s.

### Laurdan Microscopy and Generalized Polarization Calculations

Laurdan (6-Dodecanoyl-N, N-dymethyl2-naphthylamine, Sigma-Aldrich) was used to detect the liquid ordering in the membrane, as described (Bach and Bramkamp, 2013), with modifications. Cells were grown in LB until late exponential phase. Laurdan, dissolved in dimethylformamide (DMF), was added at 10 μM final concentration and cells were incubated for 10 min in the dark at 37°C, 200 rpm. Cells were then washed twice in PBS buffer supplemented with 0.2% (w/v) glucose and 1% (w/v) DMF, and resuspended in fresh pre-warmed medium. Laurdan was excited at 360 ± 20 nm, and fluorescence emission was captured at 460 ± 25 nm (exposure time: 500 ms) and at 535 ± 25 nm (exposure time: 1 s) (Strahl et al., 2014). The image analysis including the generation of GP maps was carried out using FIJI Software (Schindelin et al., 2012) in combination with the macro tool CalculateGP designed by Norbert Vischer.^1^ The overall GP values were measured for at least 100 individual cells from two biological replicates, after background subtraction.

## Data Availability Statement

The original contributions presented in the study are included in the article/Supplementary Material, further inquiries can be directed to the corresponding author/s.

## Author Contributions

AS and SB performed the all experiments and fluorescence microscopy. AS, DW, and MB conceived the study, analyzed the data, and supervised the work. All authors wrote the manuscript.

## Conflict of Interest

The authors declare that the research was conducted in the absence of any commercial or financial relationships that could be construed as a potential conflict of interest.

## Publisher’s Note

All claims expressed in this article are solely those of the authors and do not necessarily represent those of their affiliated organizations, or those of the publisher, the editors and the reviewers. Any product that may be evaluated in this article, or claim that may be made by its manufacturer, is not guaranteed or endorsed by the publisher.

## Footnotes

1
http://sils.fnwi.uva.nl/bcb/objectj/examples/CalculateGP/MD/gp.html
