# Supplementary material for: An Stomatin, Prohibitin, Flotillin, and HflK/C-Domain Protein Required to Link the Phage-Shock Protein to the Membrane in Bacillus subtilis
Source: Front Microbiol. 2021 Oct 28;12:754924. doi: 10.3389/fmicb.2021.754924 (PMC8581546; doi:10.3389/fmicb.2021.754924)

**Supplemental Material**

# *An SPFH-domain protein required to link the phage-shock protein to the membrane in Bacillus subtilis*

**Abigail Savietto Scholz[1,2], Sarah Baur[1], Diana Wolf[3], Marc Bramkamp[1, 2*]**

[1]Institute for General Microbiology, Christian-Albrechts-University of Kiel, Germany;

[2] Faculty of Biology, Ludwig-Maximilians-Universität München, Germany;

[3]Institute of Microbiology, Technische Universität Dresden, Germany.

**Correspondence:* Marc Bramkamp, Institute for General Microbiology, Christian-Albrechts-University, Germany. E-mail: bramkamp@ifam.uni-kiel.de

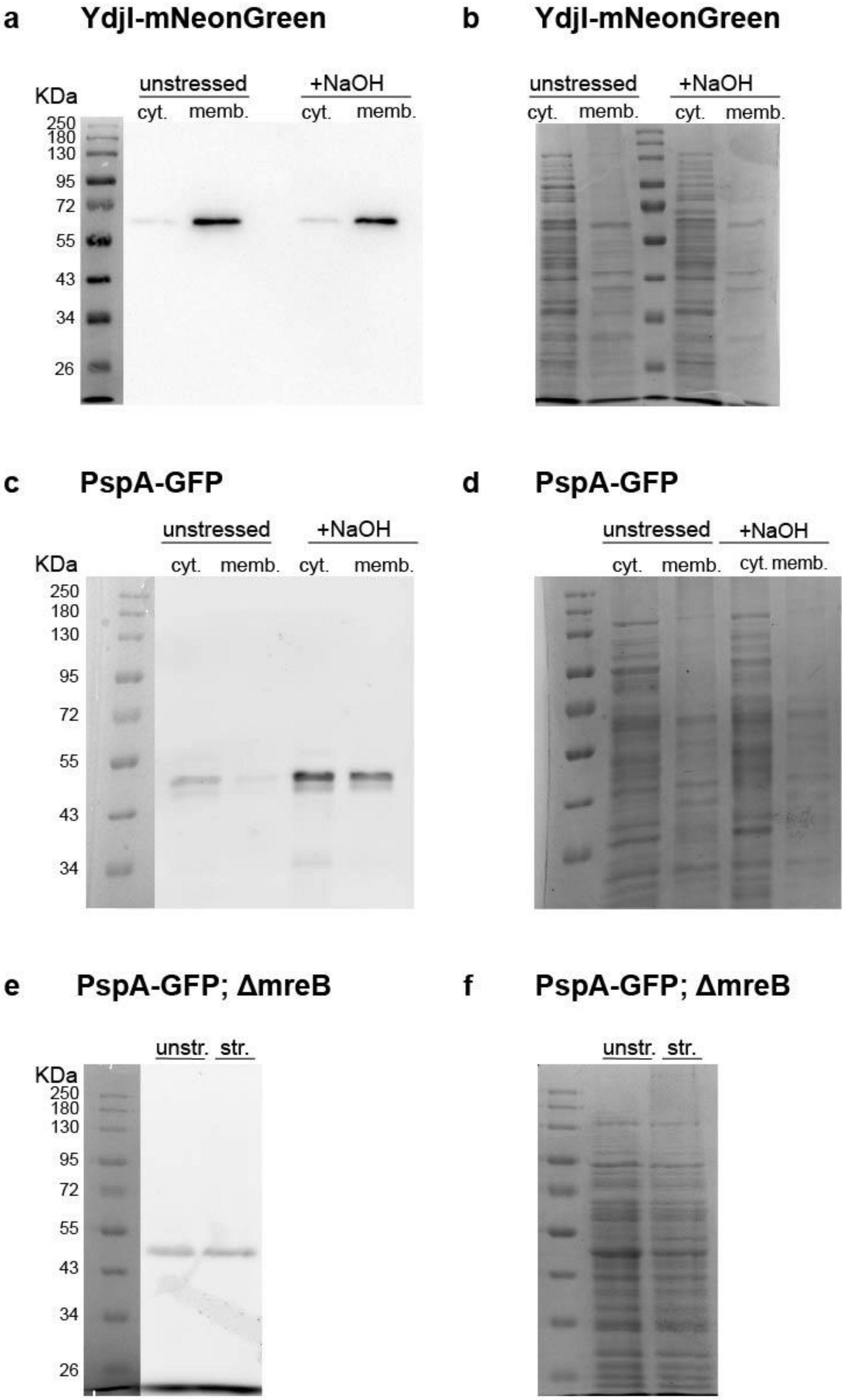

a
YdjI-mNeonGreen
unstressed
+NaOH
KDa
cyt.
memb.
cyt.
memb.
250
180
130
95
72
55
43
34
26
b
YdjI-mNeonGreen
unstressed
+NaOH
cyt.
memb.
cyt.
memb.
c
PspA-GFP
unstressed
+NaOH
KDa
cyt.
memb.
cyt.
memb.
250
180
130
95
72
55
43
34
d
PspA-GFP
unstressed
+NaOH
cyt.
memb.
cyt.
memb.
e
PspA-GFP; ΔmreB
unstr.
str.
KDa
250
180
130
95
72
55
43
34
26
f
PspA-GFP; ΔmreB
unstr.
str.

**Figure S1. (A)** Western-blot of strain YdjI-mNeonGreen (ASB033) showing cytosolic and membrane fractions under unstressed or stressed conditions (15mM NaOH). Blot was developed with HRP and excited for 90 seconds for visualization. **(B)** Coomassie staining of the corresponding SDS-PAGE gel blotted in A, as loading control. **(C)** In-gel fluorescence of SDS-PAGE containing cytosolic and membrane fractions of PspA-GFP (TSB2351), with and without stress condition (15 mM NaOH); Ex/Em: 488 nm / 526 nm. **(D)** Coomassie staining of the corresponding SDS-PAGE shown in C as loading controls. **(E)** In-gel fluorescence Δ*mreB pspA-gfp* grown with and without stress); Ex/Em: 488 nm / 526 nm. **(F)** Coomassie staining of the corresponding SDS-PAGE shown in E as loading controls.

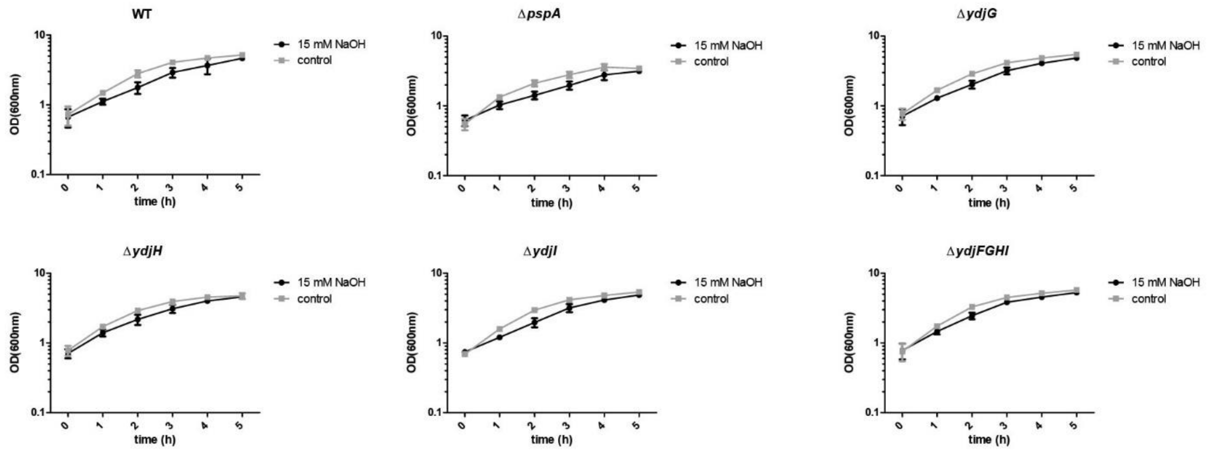


**Figure S2.** Growth curves of all mutant strains of proteins encoded *pspA-ydjGHI* operon in unstressed or alkali stress (15 mM NaOH) conditions. Error bars represent standard deviation of three independent biological replicates. No significant difference was found between the strains within the treatment and control groups ($p > 0.01$, Mann-Whitney Test).

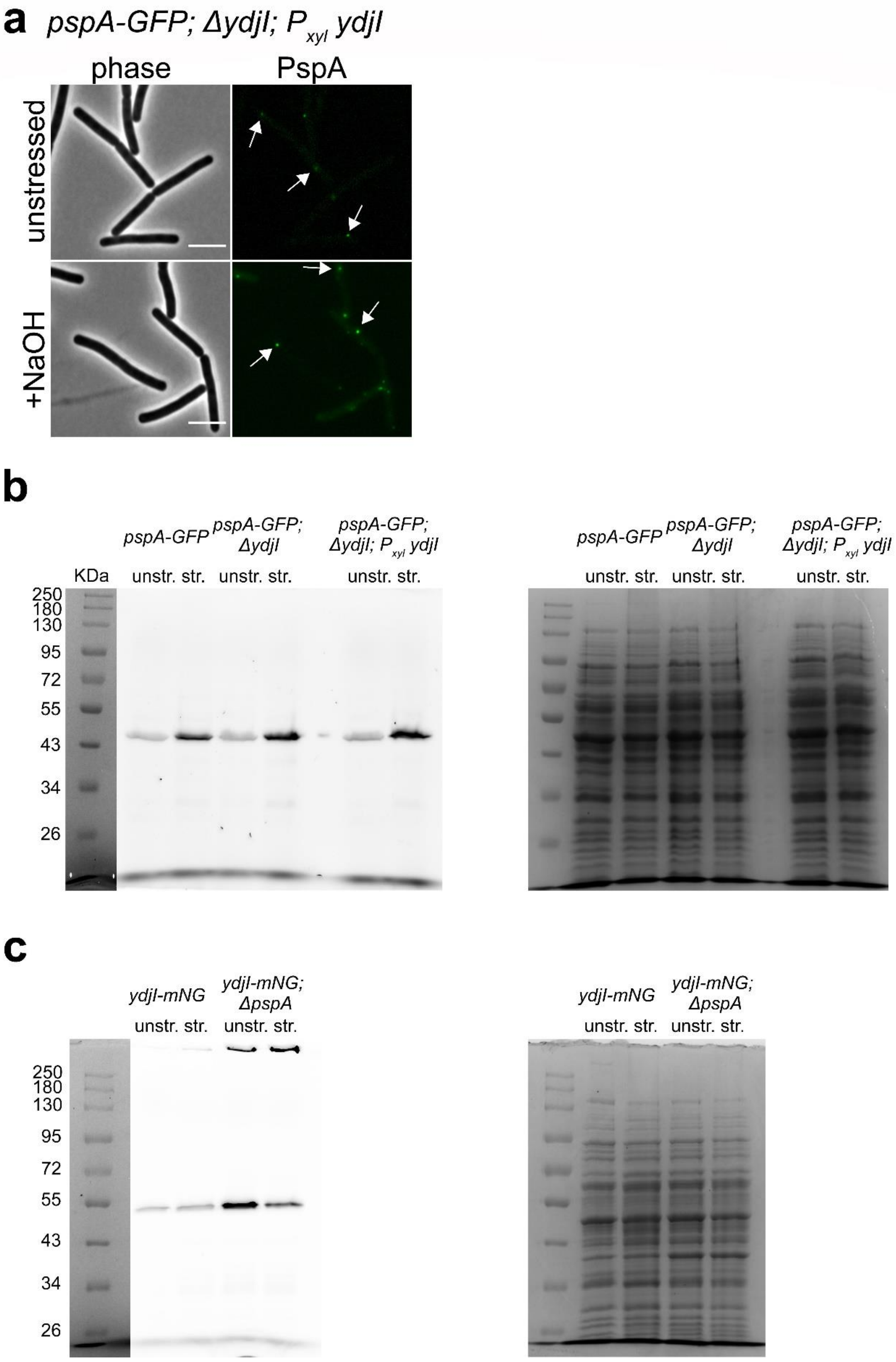
a
pspA-GFP; ΔydjI; Pxyl ydjI
phase
PspA
unstressed
+NaOH
b
pspA-GFP
pspA-GFP; ΔydjI
pspA-GFP; ΔydjI; Pxyl ydjI
KDa
unstr. str.
250
180
130
95
72
55
43
34
26
c
ydjI-mNG
ydjI-mNG; ΔpspA
unstr. str.

**Figure S3. (A)** Complementation of YdjI-mediated PspA focus formation. A *ydjI* deletion is complemented with an ectopic expression of *ydjI* from a xylose inducible promoter in a strain expressing *pspA-GFP* (strain ASB167). Cells were grown in BHI without additional xylose. This was sufficient for basal expression of the ectopic *ydjI*. Under these conditions PspA focus formation is readily complemented. Arrows point to PspA foci. Scale bars: 4 µm. **(B)** In gel fluorescence analysis of strain TSB2351 expressing *pspA*-GFP, *pspA*-GFP Δ*ydjI* (ASB162), and *pspA*-GFP Δ*ydjI* $P_{xyl}$-*ydjI* (ASB167) under unstressed or stressed conditions (15mM NaOH). A coomassie stained gel of the corresponding SDS-PAGE is shown as loading control. **(C)** In gel fluorescence analysis of strain ASB033 (*ydjI*-mNeonGreen) and strain ASB153 (*ydjI*-mNeonGreen Δ*pspA).* Note that in the strain deleted for *pspA* (ASB153) the expression of *ydjI* is upregulated. This is likely due to the loss of the Rho-independent terminator between the *pspA* and the *ydjG* genes. YdjI oligomers were observed in particular under stress conditions, as indicated by the blot signal at the top of the gel. These are apparently large protein complexes that are not denatured and do not enter the separating gel. A coomassie stained gel of the corresponding SDS-PAGE is shown as loading control.

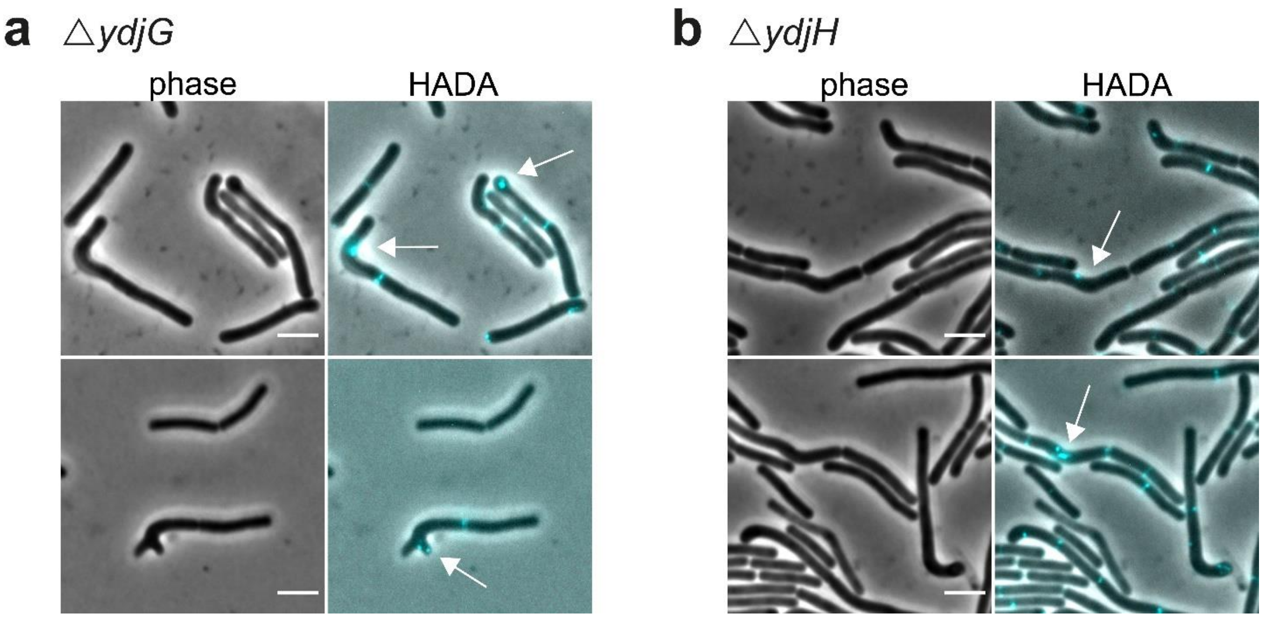


**Figure S4. Peptidoglycan synthesis machinery is delocalized upon deletion of *ydjG* and *ydjH.* (A, B)** Mutant YdjG (ASB054) and YdjH (ABS055) strains were grown in LB including 15 mM NaOH and stained with HADA as a peptidoglycan synthesis machinery probe. Exemplary images are shown in both panels. Arrows indicate miss-localization of peptidoglycan machinery and membrane cytoskeleton associated phenotype. Note that the phenotype in the *ydjG* mutant is more pronounced, indicating a stronger interaction of YdjG with cell-shape controlling machinery. Scale bars: 3 µm.

**Supplemental Material Movies**

Still images of cells shown in supplementary movies S1-8 are shown below. Cells were grown in LB medium and alkaline shock was induced by addition of 15 mM NaOH. Strains mutated in *mreB* were grown in LB supplemented with 25 mM $MgCl_2$ and 0.3 M sucrose to maintain rod-shaped morphology. For time lapse movies cells were mounted on agar pads with the indicated media and images were taken every 30 second for 5 minutes in total. Insets show the corresponding phase contrast image. Scale bar is always 2 µm.

**Movie S1.**

**YdjI-mNeonGreen (unstressed):**

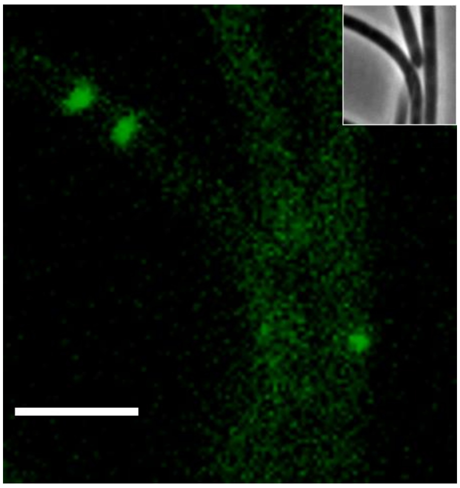

**Movie S2**

**YdjI-mNeonGreen (NaOH):**

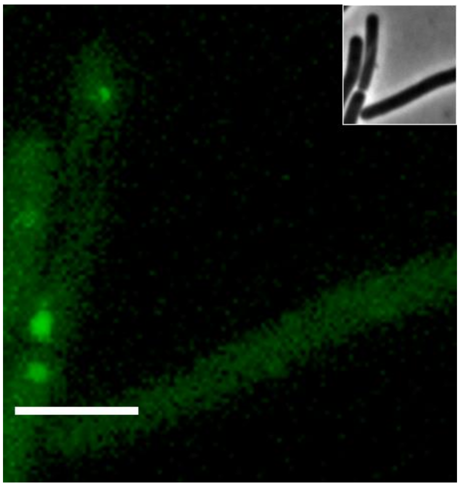

**Movie S3**

**YdjI-mNeonGreen; Δ*mreB* (unstressed):**

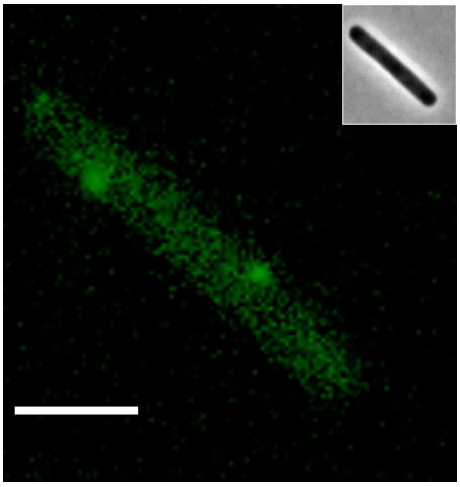

**Movie S4**

**YdjI-mNeonGreen; Δ*mreB* (NaOH):**

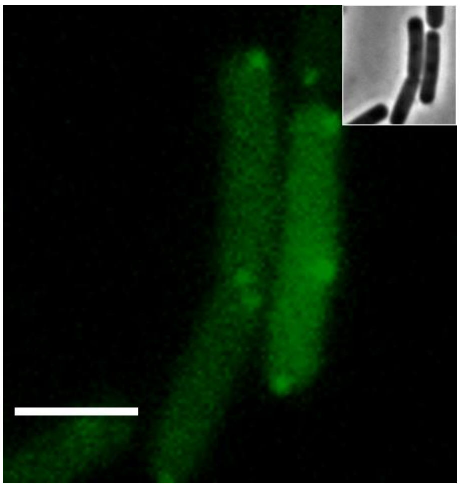

**Movie S5**

**PspA-GFP (unstressed):**

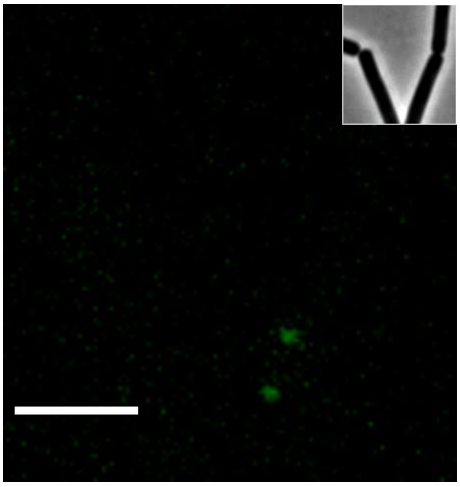

**Movie S6**

**PspA-GFP (NaOH):**

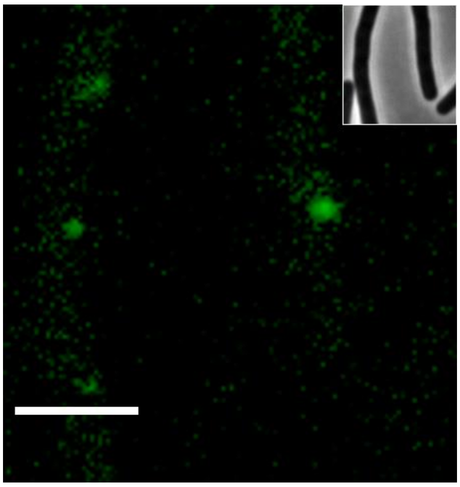

**Movie S7**

**PspA-GFP; Δ*mreB* (unstressed):**

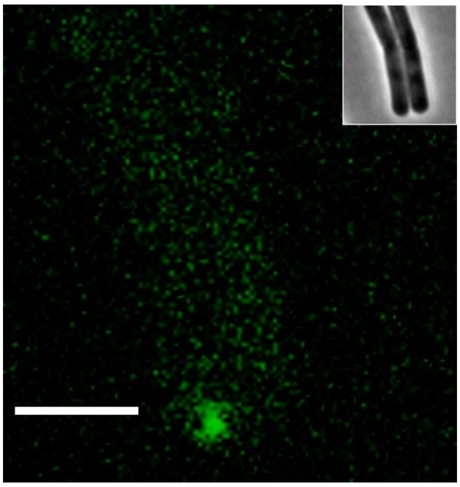

**Movie S8**

**PspA-GFP; Δ*mreB* (NaOH):**

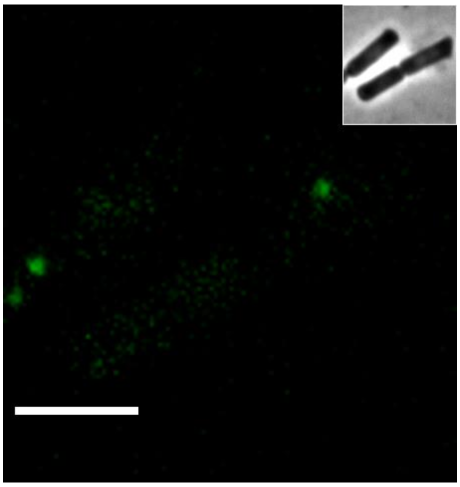

Supplement: Supplementary file 1 [file Data_Sheet_1.PDF]
