# Supplementary figures and images for: An Stomatin, Prohibitin, Flotillin, and HflK/C-Domain Protein Required to Link the Phage-Shock Protein to the Membrane in Bacillus subtilis
Source: Front Microbiol. 2021 Oct 28;12:754924. doi: 10.3389/fmicb.2021.754924 (PMC8581546; doi:10.3389/fmicb.2021.754924)

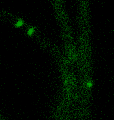

Supplement: Supplementary file 2 [file Data_Sheet_2.ZIP › Movie S1_YdjImNG control singlecell-1.gif]

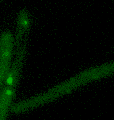

Supplement: Supplementary file 2 [file Data_Sheet_2.ZIP › Movie S2_YdjImNG NaOH singlecell-1.gif]

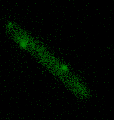

Supplement: Supplementary file 2 [file Data_Sheet_2.ZIP › Movie S3_dmreB YdjImNG control singlecell-1.gif]

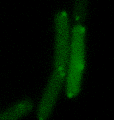

Supplement: Supplementary file 2 [file Data_Sheet_2.ZIP › Movie S4_dmreB YdjImNG NaOH singlecell-1.gif]

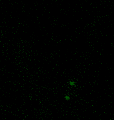

Supplement: Supplementary file 2 [file Data_Sheet_2.ZIP › Movie S5_pspA-GFP_control_singlecells-1.gif]

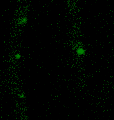

Supplement: Supplementary file 2 [file Data_Sheet_2.ZIP › Movie S6_pspA-GFP_NaOH_singlecells-1.gif]

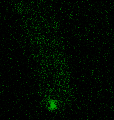

Supplement: Supplementary file 2 [file Data_Sheet_2.ZIP › Movie S7_dmreB_pspA-GFP_control_singlecells-1.gif]

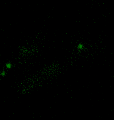

Supplement: Supplementary file 2 [file Data_Sheet_2.ZIP › Movie S8_dmreB_pspA-GFP_NaOH_singlecells-1.gif]
